# Effects of Inhaled Rosemary Oil on Subjective Feelings and Activities of the Nervous System

**DOI:** 10.3797/scipharm.1209-05

**Published:** 2012-12-23

**Authors:** Winai Sayorwan, Nijsiri Ruangrungsi, Teerut Piriyapunyporn, Tapanee Hongratanaworakit, Naiphinich Kotchabhakdi, Vorasith Siripornpanich

**Affiliations:** 1 College of Public Health Sciences, Chulalongkorn University, Bangkok 10330, Thailand.; 2 Salaya Stem Cell Research and Development Project; Research Center for Neuroscience, Mahidol University, Salaya, Nakhonpathom 73170, Thailand.; 3 Faculty of Pharmacy, Srinakharinwirot University, Nakhon-nayok 26120, Thailand.; 4 Research Center for Neuroscience, Institute of Molecular Biosciences, Mahidol University, Salaya, Nakhonpathom 73170, Thailand.

**Keywords:** *Rosmarinus officinalis* L., Electroencephalography, Alpha power, Autonomic nervous system, Mood state

## Abstract

Rosemary oil is one of the more famous essential oils widely used in aroma-therapy. However, the effects of rosemary oil on the human body, in particular the nervous system, have not been sufficiently studied. This study investigates the effects of the inhalation of rosemary oil on test subjects’ feelings, as well as its effects on various physiological parameters of the nervous system. Twenty healthy volunteers participated in the experiment. All subjects underwent autonomic nervous system (ANS) recording. This consisted of measurements of skin temperature; heart rate; respiratory rate; blood pressure; evaluations of the subjects’ mood states; and electroencephalography (EEG) recordings in the pre-, during treatment, and post-rosemary inhalation periods as compared with control conditions. Our results showed significant increases in blood pressure, heart rate, and respiratory rate after rosemary oil inhalation. After the inhalation treatments, subjects were found to have become more active and stated that they felt “fresher”. The analysis of EEGs showed a reduction in the power of alpha1 (8–10.99 Hz) and alpha2 (11–12.99 Hz) waves. Moreover, an increment in the beta wave (13–30 Hz) power was observed in the anterior region of the brain. These results confirm the stimulatory effects of rosemary oil and provide supporting evidence that brain wave activity, autonomic nervous system activity, as well as mood states are all affected by the inhalation of the rosemary oil.

## Introduction

Approximately 400 types of essential oils are currently available for therapeutic and clinical uses. They can be applied to a subject in order to induce relaxation or to reduce specific symptoms. Rosemary, scientifically labeled as *Rosmarinus officinalis* L., is a small perennial shrub of the mint family. Rosemary oil is essentially the extract of a small light blue flower along the with extract from the leaves to yield the fragrance of the essential oil, which is sometimes used as an ingredient in perfumes as well as for a food flavoring. Rosemary oil has a powerful, refreshing herbal smell with the appearance of clear water and a viscous texture [1]. Rosemary oil has been shown to be beneficial in the treatment of certain medical problems such as acne, baldness, rheumatic pain, and circulatory blockages. In addition, rosemary oil has a pronounced action on the brain and central nervous system (CNS) and is a powerful tool in helping to clear the mind and for increasing mental awareness. It has also been shown to possess excellent brain-stimulating properties as well as an aid for memory improvement [2]. The main chemical components of rosemary oil are α-pinene, camphor, and 1,8-cineole. The general properties of these substances include carminative, aromatic, antispasmodic, anti-depressant, antimicrobial, astringent, and stimulatory actions [3].

Numerous extensive studies have been done on the effects of rosemary oil. *In vitro* studies with 1,8-cineole have been reported to have shown a stimulatory effect on the cerebral cortex of rats [4]. It has been found that after exposure to 0.5 ml of rosemary oil for a period of 1 hour, the locomotive activity of mice was increased [5]. Furthermore, Graham *et al.* explored the influence of rosemary on the behavior of dogs and found that the diffusion of rosemary into the dogs' environment significantly encouraged the dogs to stand and move about significantly more when compared to exposure to other types of odoriferous substances [6]. The stimulatory effects can also be observed in human subjects. A group of 35 volunteers, after having been massaged with rosemary oil, showed an increase in the measurements of their blood pressures and breathing rates. At the same time, they were observed to be more attentive, more alert, and cheerful [7]. In addition, the electroencephalography (EEG) recordings showed a significant decrease in the power of the alpha (8–12 Hz) waves over the bilateral mid-frontal regions [8]. These findings, therefore, suggest that the decreased alpha power may be related to the increased level of alertness. However, brain wave changes in this experiment were measured in a small area by choosing only four electrodes over the frontal and parietal brain regions. There are other brain areas that have been reported to be involved from essential oil inhalation as well. This is the temporal area and it is associated with emotions [9].

Presently, the effects of rosemary oil inhalation on the autonomic nervous system (ANS) and people’s emotional state have not been properly studied. Although, a previous study has demonstrated alterations in ANS parameters after the transdermal administration of rosemary oil in humans [7]. However, there is still a lack of studies on the effects of rosemary oil on nervous system functions via inhalation. Many studies support that the mode of administration of chemicals could make a significant difference in terms of the level of the response. Take for example, the inhalation of methyl salicylate causes hypertension and convulsions; however, topical application of methyl salicylate reduces blood pressure due to the confinement of the local effects [10]. α-Santalol, the main constituent in East Indian Sandalwood, induces alertness after administration through inhalation. However, it reduces physiological arousal after being administered transdermally via body massage [11]. Our present study focuses on the effects of rosemary oil inhalation on the human nervous system. We are the first to examine the effects of rosemary inhalation on the nervous system focusing on aspects such as brain wave activity and ANS parameters which include: heart rate, blood pressure, respiratory rate, and skin temperature, as well as the assessment of mood states through comparative measures.

## Results and Discussion

In this study, we examined the effects of rosemary oil inhalation on the human nervous system. Brain electrical activity and ANS parameters (blood pressure, heart rate, respiratory rate, and skin temperature) were recorded as the indicators of the arousal level of the nervous system. We also studied the effects of rosemary oil on moods by performing subjective self-evaluation in order to assess the arousal levels.

### Autonomic nervous system parameters

Our results demonstrate significant changes in ANS parameters after exposure to sweet almond oil and rosemary oil. The data of various ANS parameters were compared during rest (control), sweet almond oil inhalation, and rosemary oil inhalation as shown in Table 1. The heart rate of subjects was significantly reduced during sweet almond oil inhalation as compared with the resting condition (*p* < 0.05). However, after being exposed to rosemary oil, heart rate, blood pressure, and respiratory rate had significantly increased (*p* < 0.01). In contrast, skin temperature had decreased significantly. The results have shown consistency with a previous rosemary oil massage study which found that rosemary oil increased blood pressure and respiratory rates [7]. The stimulatory effects on the autonomic nervous system may be explained through the abundance of oxides (1,8-cineole) and monoterpenes (α-pinene) that are present in rosemary oil. Both of these components have stimulating effects on the nervous system through sympathetic activity [12, 13]. Heuberger *et al.* found that the respiratory rate increased after the administration of 1,8-cineole [14], while Orhan *et al.* found that 1,8-cineole and α-pinene moderately inhibited acetylcholinesterase [15], an acetylcholine degradation enzyme which results in prolonged muscle contraction, and might be responsible for the reductions in skin temperatures.

### Emotional parameters

**Fig. 1:**
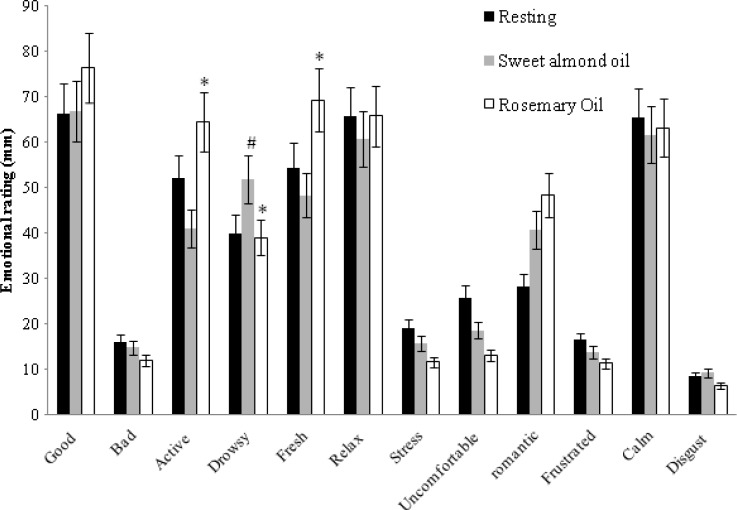
Emotional rating scores for resting condition, sweet almond oil inhalation, and rosemary oil inhalation. *p < 0.05 significance when compared with sweet almond oil inhalation, and ^#^*p* < 0.05 significance when compared with resting condition.

The alterations of mood states after being exposed to the aromas are shown in Figure 1. The participants felt fresher, became more active, and less drowsy after exposure to the rosemary oil (*p* < 0.05). Our results indicate that rosemary oil inhalation increases the level of arousal as assessed through our test subjects’ self-evaluation. All the data has collectively shown a medicinal benefit of rosemary oil when inhaled, by the removal of feelings of boredom and by providing fresh mental energy. This is in accordance to a study done by Battaglai [16]. In animal studies, rosemary vapor blended with the room air increased the level of alertness in the test dogs and encouraged them to stand and move about more when compared with exposure to lavender or chamomile [6]. Moss and colleagues assessed the olfactory impact of rosemary and lavender essential oils on cognitive performance and mood in healthy volunteers [17]. They found that rosemary produces a significant enhancement in memory performance. In regard to mood, subjects felt significantly fresher and were more alert than in the control group. Moreover, massage with the use of rosemary oil also resulted in more vigor and produced a more cheerful feeling [7]. Thus, our results confirm that rosemary oil contains mood-elevating bioactive components that prove to be beneficial to its users.

### EEG parameters

**Fig. 2:**
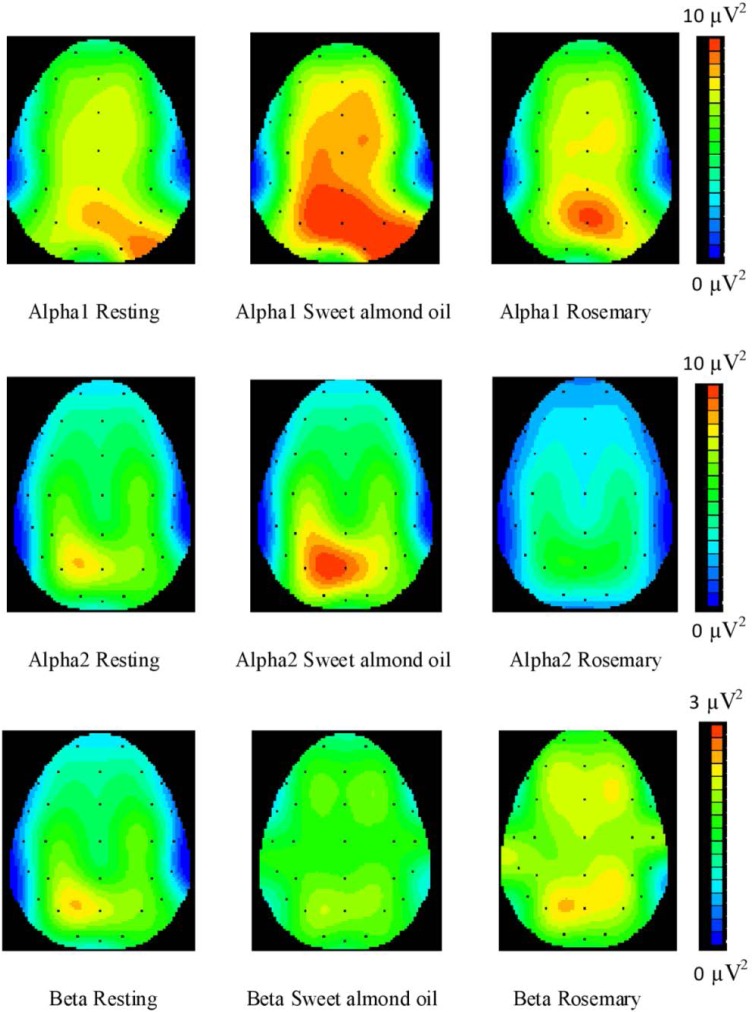
Brain topographical map of the distribution of power of alpha1, alpha2, and beta activities for the resting condition, sweet almond oil inhalation and rosemary oil inhalation.

In EEG analyses, the power (amplitude^2^) was calculated for each frequency band (delta, theta, alpha, alpha1, alpha2, and beta) during rest, during sweet almond oil inhalation, and during rosemary oil inhalation states. The areas of interest were grouped into the left anterior area (Fp1, F3, F7), the right anterior area (Fp2, F4, F8), left posterior area (P3, T5, O1), right posterior area (P4, T6, O2), and the central (FCz, Cz, CPz) brain regions [18, 19]. During the sessions of rosemary inhalation, the power of the alpha1 waves in the left and right anterior and right posterior regions was significantly decreased (*p* < 0.05). The power changes in the alpha2 waves were also significantly decreased during rosemary inhalation as was shown in all areas of the brain (*p* < 0.05) (Table 2). In contrast, the power of the beta waves in the left and the right anterior brain regions was significantly increased (Table 3). No significant changes were observed in the case of the theta wave’s power (*p* > 0.05, data not shown). The brain’s topographical map demonstrated less spreading of the alpha1 waves in the frontal area and the right posterior brain region during rosemary oil inhalation as compared to sweet almond oil. The topographic map of the alpha2 waves has shown reduced spreading of these waves in all brain regions. In contrast, the power of the beta waves in the frontal area had shown an increase (Figure 2).

The present study shows the clear effects of rosemary oil inhalation on brain waves. During inhalation of rosemary oil, the power of alpha1 (8–10.99 Hz) and alpha2 (11–12.99 Hz) activities considerably decreased, however, the power of beta waves (13–30 Hz) significantly increased in the frontal area. These results show concordance with the pattern of EEG variation with the arousal level of the CNS. The increase in central activation is typically characterized by a decrease in alpha activities but with an increase in beta activities [20]. Our findings regarding the decrease in frontal alpha1 and alpha2 power after rosemary oil inhalation is similar to the results noted in Diego’s study, but also differ in terms of the beta wave changes [8]. Increased beta activity in our study can be explained by the enhanced alertness, which is advantageous for thinking processes. The beta wave is thought to be related to the discrete neuronal activities on the cortical surface of the brain. Thus, increasing beta power over the frontal region, the brain area for higher cognition, may be related to the thinking process. Furthermore, previous studies support that 1,8-cineole, the main constituent in rosemary, has exhibited an impact on brain waves. For instance, Nakagawa *et al.* found that methyl jasmonate and 1,8-cineole in jasmine oil can increase beta waves and inhibit the enhancement of alpha and theta waves that corresponded to stimulating effects to the brain [21]. As a result, 1,8-cineole, may be involved in the underlying mechanism of the effects of rosemary oil on the CNS in our study.

## Conclusion

In brief, our results suggest the occurrence of the positive stimulatory effects of rosemary oil inhalation. These findings provide evidence that brain wave activities, autonomic nervous system responses, and mood states can all be modified with rosemary oil inhalation. The results support that there are medicinal benefits of rosemary when used as a stimulant in essential oil treatments.

## Experimental

### Subjects

A total of 20 healthy subjects (10 males and 10 females), ages between 18 to 28 years (mean age 21 ±2.97 years) and body mass index (BMI) between 20–25 kg/m^2^ (mean BMI 20.69 ± 1.69 kg/m^2^) were enrolled in this study. We had taken into consideration that obesity can affect the parameters of the autonomic nervous system’s measurements [22]. All experimental procedures were followed with the strict ethical standards formulated in the Helsinki Declaration of 1964 that was revised in 2000 and were approved by the Ethical Review Committee for Research Involving Human Research Subjects, Health Science Group, Chulalongkorn University and Mahidol University Institutional Review Board (MU-IRB). All the steps in this experiment were similarly conducted as per the previous study recorded on the effects of lavender oil inhalation [19]. Handedness was assessed by the Edinburgh Handedness Inventory scale and only right-handed subjects were selected [23]. The subjects were then screened for a normal sense of smell by using the n-butyl alcohol method test [24] (mean score 9.65 ± 0.96). Personal health status was also recorded. This included weight, height, and blood pressure. All of the subjects who passed the screening procedure were non-smokers [25], did not exhibit any symptoms of upper respiratory infections, were absent of neurological diseases, and did not have a history of hypertension or cardiovascular disease. Female subjects were not to be in their menstrual period on the day of the tests [26]. All subjects should not be showing signs of fatigue or drowsiness before the start of the experiments.

### Essential oil preparation and administration

The composition of rosemary oil obtained from the Thai China Flavors and Fragrances Company was analyzed by gas chromatography/mass spectrometry (GC/MS) equipped with the Finnigan DSQ MS detector, Thermo Finnigan model Trace GC Ultra. The oil’s constituents were identified by matching their mass spectra and retention times with the compounds indicated in the NIST05 MS library. The percentage of compositions was computed from the GC peak areas. The results revealed that rosemary oil is composed mainly of 19.41% α-pinene, 20.08% 1,8-cineole, and 21.25% camphor. One milliliter of sweet almond oil (base oil) or 10% v/v rosemary oil, diluted with a base oil, was administered using an oxygen pump connected with a respiratory mask through a plastic tube (adult inhalation set) with the airflow rate set at 2 liters/min. In accordance with previous studies, it has been found that the pleasantness of the aroma of the oil could alter autonomic activity [27, 28]. As a result of these facts, the subjects were asked to inhale base oil and rosemary oil to assess the level of the pleasantness experienced by using a 5-point Likert scale prior to starting the experiments. The subjects with the rates of pleasantness that were within 2–4 were allowed to proceed in the experiments.

### Acquisition of autonomic parameters and mood state

The mood states and ANS parameters of the subjects such as: blood pressure, heart rate, skin temperature, and respiratory rate were recorded simultaneously. The ANS parameters were recorded using the Life Scope 8 bedside monitor (Nihon Kohden, Japan), while the Geneva Emotion and Odor Scale (GEOS) was used for mood state ratings [29]. This particular scale consisted of a 100 mm monopolar visual analog scale following five mood states: pleasant (good), unpleasant (bad, uncomfortable, disgusted, frustrated, and stressful), sensual (romantic), relaxed (relax, calm, drowsy), and refreshed (fresh, active).

### Acquisition of the electroencephalographic data

A set of 31 electrodes with an additional ground electrode were placed onto the subject’s head in accordance to the international 10–20 system at FP1, FP2, FZ, F3, F4, F7, F8, FT7, FC3, FCZ, FC4, FT8, T3, T4, T5, T6, TP7, TP8, C3, CP3, C4, CZ, CPZ, CP4, P3, P4, PZ, O1, O2, and OZ. The average of both mastoid areas (A1+A2)/2 was used as the recording reference. The electrooculogram (EOG) was measured by placing four electrodes in two external acanthi (HEOL and HEOR), the left supraorbital (VEOU), and the infraorbital (VEOL) regions. An Electro-Cap was made of an elastic spandex-type fabric with silver/silver chloride (Ag/AgCl) electrodes attached to the fabric. Electrode impedances were adjusted to lower than 5 kOhms. Acquire Neuroscan version 4.3 (Neurosoft, INC) was used as the recording system. An online filter was set to band pass; with a low frequency of 70 Hz and a high frequency of DC. Analog-to-digital (A/D) rate was set at 500 Hz, the gain was set at 19, and the notch filter was set at 50 Hz. The relative power spectrum of the respective frequency bands derived by Fast Fourier Transformation (FFT) was expressed as follows: Delta (0–3.99 Hz), Theta (4–7.99 Hz), Alpha (8–12.99 Hz), and Beta (13–29.99 Hz) wave ranges [30, 31]. Furthermore, the alpha wave was further categorized as alpha1 or low frequency alpha (8–10.99 Hz) and alpha2 or high frequency alpha (11–12.99 Hz) activities.

### Experimental protocol

The experimental protocol has been previously set by our group [19]. Experiments were conducted in the morning hours only (8.00–12.00 AM) in order to reduce circadian variation. All activities were done in a silent room with an ambient temperature of 24±1°C and at 40–50% humidity. The ANS electrodes were attached to accommodate suitable positions after the subjects were seated comfortably in the adjustable armchair. Heart rate, skin temperature, and respiratory rate were recorded every minute, while the systolic and diastolic blood pressures were recorded every 5 min. The experiment was divided into three sessions, the first portion served as a 10 minute baseline trial (resting period). The second and the third trials took 20 min each. Sweet almond oil was administered in the second trial, where 10% (v/v) rosemary oil diluted in sweet almond oil was exposed during the third trial. Subjects were required to rate their mood states at the end of each trial.

The EEG experiment was similar to those done in the ANS experiments. The procedure was divided into three sessions of 7 min each. However, resting (baseline) EEG recordings were done separately with both eye-closed and eye-opened sessions. Following these, the participants were exposed to sweet almond oil and then 10% v/v rosemary oil with 5 minute intervals.

### Statistical analyses

The SPSS statistical package version 17 was used for data analyses of the effects of rosemary on physiological reactions and mood states, before and after the rosemary inhalation. A paired t-test was carried out on data concerning the subjects’ blood pressures, heart rates, skin temperatures, powers of the brain waves, and their ratings of their mood states. A non-parametric Wilcoxon signed rank test was used for respiratory rate analysis.

## Figures and Tables

**Tab. 1:** Mean and standard deviation of ANS parameters for resting condition, sweet almond oil inhalation, and rosemary oil inhalation.

**ANS parameters (n=20)**	**Resting**	**Sweet almond oil**	**Rosemary oil**
Systolic BP	105.40 ± 8.54	105.71 ± 8.57	108.31 ± 8.88^**^
Diastolic BP	64.26 ± 11.04	63.93 ± 5.80	70.17 ± 7.97^**^
Heart rate	71.97 ± 11.19	69.43 ± 9.52^*^	72.25 ± 10.22^**^
Skin temperature	32.12 ± 1.84	32.24 ± 1.97	31.79 ± 1.88^**^
Respiratory rate	15.98 + 1.96	15.72 + 2.55	16.58 + 2.65^**^

* *p* < 0.05 significance when compared to resting condition, and

** *p* < 0.01 significance when compared to sweet almond oil.

**Tab. 2:** Mean power values of alpha1 and alpha2 waves for resting condition (eye-closed recording), sweet almond oil inhalation and rosemary oil inhalation.

**Brain area**	**Average alpha 1 power (μV^2^)**		
	**Resting (eye-close)**	**Sweet almond oil**	**Rosemary oil**
Left anterior	5.25	6.35	5.42^*^
Right anterior	5.39	6.58	5.57^*^
Central area	8.56	10.15	8.74
Left posterior	6.87	7.80	6.01
Right posterior	8.03	9.59	6.88^*^
**Brain area**	**Average alpha2 power (μV2)**		
	**Resting (eye-close)**	**Sweet almond oil**	**Rosemary oil**
Left anterior	2.71	2.72	2.20^*^
Right anterior	2.68	2.63	2.14^*^
Central area	4.32	4.60	3.62^*^
Left posterior	4.77	5.30	3.57^*^
Right posterior	5.30	5.82	3.98^*^

* *p* < 0.05 significance when compared with sweet almond oil.

**Tab. 3:** Mean power values of beta wave for resting condition (eye-closed recording), sweet almond oil inhalation, and rosemary oil inhalation.

**Brain area**	**Average beta power (μV^2^)**		
	**Resting (eye-close)**	**Sweet almond oil**	**Rosemary oil**
Left anterior	0.29	0.31	0.36^*^
Right anterior	0.31	0.31	0.37^*^
Central area	0.38	0.39	0.42
Left posterior	0.35	0.37	0.37
Right posterior	0.33	0.38	0.36

* *p* < 0.05 significance when compared with sweet almond oil.
